# Online Feedback Systems and Debate on Scientific Issues during COVID-19: A Case Study on Sookmyung Women’s University

**DOI:** 10.3390/ejihpe12050037

**Published:** 2022-05-23

**Authors:** Ji-yoon Kim, Min-kyeong Shim, Young-mee Hwang

**Affiliations:** 1 General Education Research Institute, Sookmyung Women’s University, Seoul 04310, Korea; vantablack@sookmyung.ac.kr; 2 Department of Environmental Design, Sookmyung Women’s University, Seoul 04310, Korea; mkshim@sookmyung.ac.kr; 3 School of General Education, Sookmyung Women’s University, Seoul 04310, Korea

**Keywords:** COVID-19 pandemic, university education, discussion programs, online discussion clinic, online discussion feedback systems, educational guidelines, smart learning

## Abstract

As the COVID-19 pandemic continues, university education and feedback guidance have inevitably moved to online platforms, becoming a global trend. This study focuses on a case of Sookmyung Women’s University in South Korea, which has operated an online discussion clinic for university general education for more than a year as a case study. There are two main research methods. A frequency analysis was conducted to confirm what kind of counseling the students preferred at the discussion clinic based on the answers written in the students’ applications. The students whose applications were used for the analysis were divided into 57 teams, and there were two to six members per team. The results were as follows: In the survey results, students wanted help with the preparation process necessary for the discussion and the practical strategies for facilitating discussions. They wanted personalized counseling, demonstrating that discussion education provided in the foundational curriculum is insufficient. Second, the educational model of the discussion clinic and educational examples were examined. The findings confirmed that online discussion education is effective if the system is technically supplemented. Instructors and researchers are prepared to meet students’ demands for feedback and individual counseling, even if these are not provided through face-to-face discussions. Additionally, face-to-face guidance can be operated more effectively by taking advantage of online systems. The findings also demonstrate that further research on designing and operating online discussion centers is required. This study is a preceding study on developing online systems and educational guidelines for higher educational institutions to present new insights into smart learning. This paper also includes suggestions for educational and scientific discussions. The online discussion instructional model shown in this paper explores methods of scientific communication through a debate on scientific issues.

## 1. Introduction

University discussion classes in South Korea have continued to develop since the late 2000s [1], “when the need for a humanities-centered curriculum was pointed out” [2]. The importance of communication in university education is related to the increasing demands of the times that emphasize the importance of speaking as an active subject and the increasing value of speaking skills in the job market or work environment [3]. 

Litan [4] argued that we are now more divided politically and economically than ever and that COVID-19 has further accelerated this division. According to Litan [4], we have gone beyond simple polarization, and instead we have become “tribal,” meaning that whatever we say or write, we will always be viewed by others from divided perspectives. He found a prescription for these problems in the “incorporation of debate of evidence-based argumentation” [4] in the educational field, emphasizing the need for debate-centered education.

In many South Korean universities, discussion classes as part of liberal arts and general education are facilitated as part of the curriculum, and education to develop the communication skills of students through presentations and discussions is widely established. Discussion-related subjects may be newly created or systematized or supplemented with existing subjects. However, although many discussion classes are taking place in universities, the proportion of discussion practice in classes is low. Additionally, as there are often many students in one class, it is difficult for a teacher to provide individual guidance in one semester. In the case of university writing classes, various counseling programs between professors and learners have been conducted separately within or outside the class to enhance the educational effect. In contrast, students participating in discussions do not have opportunities to receive professional counseling at a personal level. Although students need continuous and systematic guidance from experts, the university does not have the infrastructure to perform it.

Due to the awareness of this problem, speaking counseling institutions have recently emerged in some South Korean universities. As the number of institutions is still very small and is less than that of writing counseling institutions, it can be said that it is still at its initial stage, but it is gradually increasing. Most of them are operated as part of undergraduate advancement projects or educational competency enhancement projects, and some universities support this program with their school expenses. These university institutions run various non-curricular activities such as debate contests and presentation competitions and counseling to help students improve their public speaking skills.

Currently, universities that operate speaking clinics in South Korea include Changwon University, Sangmyung University, Daegu Catholic University, and Mokwon University. They are commissioned by educational institutions such as teaching and learning support centers and liberal arts universities to develop various learning programs. Yet, their focus is on communication rather than discussions. Some university writing clinics teach how to write introductory discussion books, but speaking is not conjoined. Therefore, Sookmyung Women’s University’s discussion clinic has a unique status because it provides a specialized program or an institution in charge of in-depth discussion education as an official center.

There are programs or clinics for speaking counseling, including discussions. However, discussion clinics as separate educational institutions are rarely established, and it is difficult to find institutions that provide personal counseling and feedback on discussions through online centers worldwide. Therefore, the case of Sookmyung Women’s University’s “Online Discussion Clinic” (ODC) in South Korea needs to be academically illuminated and shared.

With the prolonged COVID-19 pandemic, the university’s oral communication education and feedback guidance were forced to take place by establishing an online system. It was not only in South Korea but also around the world that it moved to “an alternative education delivery models and pedagogical practices in educational institutions.” [5] The key to managing student anxiety caused by the pandemic is to provide a safe and supportive school environment [5]. In Korea, Internet information technology is developing, and the interest in smart learning is increasing, so the informatization of university classes has accelerated as a way to respond to COVID-19.

The case study of Sookmyung Women’s University’s ODC covered in this article contains the educational experience of a discussion clinic operated online for a year beginning in 2020 to determine the students’ needs and their problems. This study is a preceding study on developing online systems and educational guidelines for debate to present new insights into smart learning.

Sookmyung Women’s University’s ODC differs from other institutions as it includes a counseling institution focusing on discussion, which is challenging to learn in theoretical classes. In discussion education, speakers need to process logical thinking into verbal expressions and sequence what they have studied logically.

It is important to practice verbal and non-verbal content together in the discussion [6], including the use of appropriate terminology, clear and concise voice volume and speed, clear and succinct pronunciation, delivering a speech in consideration of the audience, using appropriate language content, the attitude and passion of the speaker, good posture and presentable clothes, positive body language, and adequate use of space. These skills can only be taught and commented on during individual customized counseling classes. This is because there are very few times when professors can personally correct problems or fill in shortcomings in classes with many students.

Additionally, discussion clinics where students request what they want and receive customized education become student-led rather than teacher-led, which is often seen in usual classroom settings. In this way, education through customized counseling can achieve richer and higher-quality interactions, making it a proper way for students to attribute meaning when they learn. It also fosters communication skills through interaction and cultivates competencies necessary for actual discussions. This can be said to be constructivist. According to Vygotsky, one of the constructivists, the process of forming individual knowledge takes place through interaction with others before being internalized by individuals [7].

In this way, education through customized counseling can achieve richer and higher-quality interactions, making it a valid way for students to attribute meaning when they learn. It also fosters communication skills through interaction and cultivates competencies necessary for actual discussions.

In this article, the achievements, problems, and directions of this clinic are explored through counseling materials and examples from Sookmyung Women’s University’s ODC. The analysis results and development plans can help establish online discussion clinics and provide evidence for general discussion education regardless of the subject.

This article focuses on discussing scientific issues related to scientific thinking based on the classical definition by Russell [8] that science is organized to achieve deep and contemplative thinking to solve problems. As it is essential to inform others about new scientific achievements and convey opinions on scientific facts or scientific issues, the ability to discuss science is a necessary competency. The democratic process of establishing social, ethical standards to introduce new technologies and reach a consensus with the community serves as a legitimate reason for determining the direction of scientific research [9] (p. 299). Effective scientific communication should allow groups of experts and the general public to discuss with each other freely. Scientific communication can be enhanced through discussion education.

By presenting examples of the effectiveness of the individual discussion counseling system while conducting discussions dealing with scientific issues, this article demonstrates the necessity and utilization of developing discussion clinics and provides best practice examples for developing and implementing these forms of communication education. Studies have shown that communication education closely relates to improving critical thinking ability [10]. Discussion education encourages critical thinking and problem solving.

In the US, there have been individualized discussion counseling programs for decades. These programs have enhanced students’ ability to engage in discussions by providing individual coaching and guidance. They are often provided as an educational program for debate contests or forensics programs (comprehensive speech contests) inside and outside universities or as an educational program within universities to develop academic presentation skills. Some schools operate “Discussion Centers” as affiliated educational institutions with separate spaces on campus. In the US, there is a consensus on the importance of discussion, and accordingly, programs or institutions in charge of individual discussion education are often established in universities. One example is Houston University’s Speech and Debate Program. This program is operated as a joint curriculum program at the Honors College and allows undergraduate students to study, discuss, and foster leadership on modern society’s significant challenges. In addition to competition between universities, the program hosts open discussions, speeches, and discussion topic activities. It is not a one-time program for debate competitions and encourages campus members to participate by promoting competitive debates among colleges and hosting open discussions, speeches, and other discussion topic activities. Coaches of the teams belonging to the Department of Leadership Research also conduct courses on public policy, leadership, and other subject matters [11].

Some schools have set up separate institutions to embed basic discussions and speeches in their regular courses. The Sookmyung Women’s University’s ODC is an example of this case, and another example is Luther College, located in the US. The school has been operating the Speech and Debate Center since 2012 and serves as a tutoring center for students who want to improve their presentation skills for classes. They provide the Honors Hibbler Debate Scholarship and award USD 1000 to the debate winner every year [12].

Schools that operate discussion centers aim to help students with their university classes and become better at giving academic presentations. These programs or centers have trained senior communication majors or experienced discussion coaches who have trained in related fields and provide expert advice in preparing and delivering speeches necessary for debate competitions or college classes.

However, in the case of universities in Asia, there are few programs or institutions that nurture discussion education at universities, and it is not easy to find an example of a discussion center, despite the growing consensus that discussions need to be revitalized to break from the influence of a Confucian culture that often silences free speech. Individual discussion education is also rarely conducted in Asia. Although the number is not large, the examples of university-level discussion education conducted in Korea and China are as follows.

In the case of the speech and discussion association of Zhejiang University of China, their classes consist of an integration of the speech and the discussion teams, and they teach the skills and practice of speech and discussion. This includes delivering speeches and discussions, drafting speeches and discussions, logic and thinking, presentations, and observation of discussions. It also organizes competitive discussions within universities, such as the “Economic Management College Freshman Speech Contest” and “Economic Management College Freshman Debate.” Many private universities and national and public universities in Japan conduct group discussions as part of entrance examinations, but it is still difficult to find university general education or liberal arts courses, separate institutions, or programs. Instead, there are small discussion groups or job preparation programs to prepare for group job interviews.

In South Korean universities, discussion education is often included in core general education subjects, and discussion education is conducted in various ways. Institutions and programs that provide individualized education for discussion are also increasing. At Changwon University, operation aims to improve students’ communication capabilities by facilitating one-to-one customized online and offline counseling and improve their communication skills in writing and speaking. Additionally, a professional guest counseling institute was appointed to establish a regular communication counseling system at the General Education Center.

In the case of speaking counseling at the Catholic University of Daegu (no appointment required), the content of the counseling is related to speaking, mainly dealing with public/private speaking, and one noticeable difference from other university programs is that research professors are stationed on campus. Speaking learning materials are always accessible, and this contributes significantly to the flexible and smooth running of the counseling, as any educational material is provided immediately at universities on request.

Looking at Sangmyung University’s Communication Skills Development Center, there is a program that allows students to receive customized guidance from a professor who proofreads and examines students’ work at the Speaking Clinic. Additionally, students can attend the Speaking Clinic for training on self-introduction presentations. Individual feedback is provided to improve practical speaking skills such as PowerPoint template data, verbal and non-verbal expressions, presentation attitudes, and job interviews.

Mokwon University Speaking Clinic was established to improve speaking skills and enhance liberal arts language as part of the Advancement of College Education and operates the Mokwon Stocks Contest and Presentation Contest.

As discussed above, several universities run speaking clinics in South Korea and comparative programs, such as providing individual counseling tailored to student speaking contests. However, the focus is mainly on speech-oriented education, such as making presentation materials for college class presentations, improving presentation skills, and preparing for job interviews, and it is difficult to find a speaking clinic that specializes in discussion. Despite the increasing number of speaking clinics in South Korea, there are few existing studies in South Korean education on speaking clinics except for the study by Kang at Mokwon University [13] and the study by Shim at Seowon University [14]. Shim [14] examines the Seowon University Writing Clinic and Speaking Clinic simultaneously, so the proportion of discussion education is insignificant, and it is instead more of a review of web-based clinic systems.

Kang [13] explored Mokwon University’s comparative program linked to speaking and communication to understand the speaking clinic’s operation status, counseling procedures, methods, and characteristics in detail. Kang’s research focuses on the operation status from the second semester of 2015 to the first semester of 2017 and demonstrates that Mokwon University Speaking Clinic was established to improve undergraduate students’ speaking skills and enhance liberal arts language. It provides one-on-one feedback through a speaking education manual and speaking activities. However, as described above, it is difficult to find a center that specializes in discussion education, such as a discussion clinic operated by Sookmyung Women’s University in South Korea.

Sookmyung Women’s University’s ODC was established in the first semester of 2020 and is a counseling institution that focuses on discussion and discussion practice. In 2020, the time frame of the case study in this article, Sookmyung Women’s University’s ODC operated as a comparative program linked to the curriculum, and only students taking the Critical Thinking and Discussion course could apply. Currently, the target is being expanded, and the discussion clinic is facilitated online. After applying for a discussion clinic through the General Education Research Institute website, a consultation (40 min to 1 h) is conducted in the zoom conference room by adjusting the time with the counseling professor. Students can apply by uploading clinic content documents (introductory and discussion briefs) and counseling application forms onto the ODC website.

An appointment must be made in advance online to use the discussion clinic’s educational service, and the process is illustrated in Table 1. When students who want a discussion clinic download the application form on the website and submit it to the online bulletin board, the instructor will contact them directly, set the appointment time, and proceed to the Zoom meeting at a fixed time.

**Figure 1:**
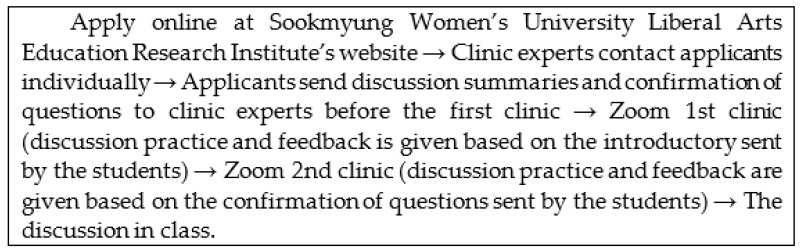
Sookmyung Women’s University Online Discussion Clinic.

Students who have completed the first and second clinics can move on to engage in discussions in class with the contents that they acquired from the clinic. As evident from Figure 1, the first round focuses on the introduction, and the second round focuses on the confirmation of questions along with completing the introduction. The system, area, and service contents of the discussion clinic are listed in Table 1.

## 2. Research Subjects and Methods

### 2.1. Research Subjects

This study targets students taking the introductory general education class Critical Thinking and Discussion (not held in 2020 due to COVID-19), and the subjects were liberal arts students who received discussion clinics for a total of two semesters from the first semester of 2020 to the first semester of 2021 at Sookmyung Women’s University. The University is a prestigious university located in Seoul and has held annual textbook discussion competitions since 2002. Discussion clinics related to the Critical Thinking and Discussion class have been piloted since 2019.

### 2.2. Research Methods

There are two main research methods. First, a frequency analysis was conducted to confirm what kind of counseling the students preferred at the discussion clinic based on the answers written in the students’ applications.

To discover patterns in qualitative data, the researchers tried to find frequencies based on the answers submitted from the students’ applications. A frequency analysis was performed by processing multiple responses, and SPSS 22 was used as an analysis tool.

The students whose applications were used for the analysis were divided into a total of 57 teams, and there were two to six members per team. In total, 119 cases had multiple responses, which accounted for 208.8% of the teams (*n* = 57). Second, the educational model of the discussion clinic and educational examples were examined.

The method of analyzing the students’ opinions in this discussion clinic largely follows Toulmin’s argument [15], who viewed arguments as a series of results in which arguments and inferences were interconnected. In other words, Toulmin defined a proven argument as a continuous cycle of inferences that are substantiated by the consolidation of an argument followed by the provision of logical reasons and evidence. His interest in the proven argument was whether the argument was accepted by others in recognition of its legitimacy only when evidence supporting the argument was presented. Toulmin’s model consists of elements that must be presented for justification of the argument. The debate education in university classrooms needs to be conducted through a systematic thinking process with legitimate arguments for the purpose of fostering critical thinking. Education that improves thinking skills is centered on students. However, the concrete thinking processes students use in their classroom are still teacher-centered. Toulmin’s model suggests a way to overcome such an educational reality [16].

The elements of Toulmin’s argument are data, arguments, and grounds in the first tier and support, disproof, and limitation in the second tier. Each element is as follows.

1. Data: Each argument is supported by data, and data are a description of facts consistent with the background. These facts are accepted as truth and depend on the previous argument and its clarity.
2. Claim: This is the argument’s conclusion and is supported by data or factual premises.
3. Warrant: This process gives validity to the process of proceeding from data to arguments.
4. Backing: This refers to the supporting action that enables the consolidation of authority for justifiable reasons.
5. Rebuttal: This refers to a situation in which the general authority of justifiable reasons is not recognized.
6. Qualifier: This is a modifier that limits the degree of certainty by attaching it to the front of an argument.

The clinic for the confirmation of questions was examined using Thyer’s “Steps of Critical Thinking Process” [17]. While there are many critical thinking theories, Thyer’s model is considered to have the most logical diversity concerning the discussion in the educational field and contains the necessary content for the critical thinking process [18].

To present an example of discussion clinic education, the students’ opinions and confirmation questions examined in this presentation were narrowed down to investigate the following scientific statements: “Should animal testing be banned” and “Paris Climate Change Convention is effective.”

## 3. Research Results and Discussion

### 3.1. Frequency Analysis Results

Based on the answers submitted in the students’ applications, a frequency analysis was conducted to confirm what kind of counseling the students preferred at the discussion clinic, and the results are as follows. For analysis, we used the SPSS software 22.0 program.

The areas in which students want to be educated at the clinic are presented in the order of their preferences: introduction, 28.6% (*n* = 34); discussion facilitation, 20.2% (*n* = 24); analysis of argument points, 18.5% (*n* = 22); discussion outline, 17.6% (*n* = 21); argument cards, 8.4% (*n* = 10); data research and organization, 5.9% (*n* = 7); and others, 0.8% (*n* = 1).

The results demonstrate that students most needed counseling on how to write introductions and want to learn discussion strategies by receiving counseling on discussion facilitation. As the need for data investigation and organization is relatively low, it can be assumed that students think they can research and organize data independently without receiving help. The results of the frequency analysis are illustrated in Table 2.

As illustrated in the survey results, students wanted help with the preparation process necessary for the discussion and the practical strategies for facilitating discussions. Although the students had already taken discussion courses, they wanted additional counseling, demonstrating that discussion education provided in the foundational curriculum is insufficient. Given that they experience hardship in the preparation process, there is a need for lectures on the introduction and how to implement discussions.

### 3.2. Education Related to Setting a Thesis and Preparing a Thesis Analysis Table

A thesis should be written with a tone of affirmation to avoid confusion in the argument. If a thesis sentence is written in negation, the opposite becomes positive due to a double negation, which can interfere with the audience’s comprehension and judgment. This interruption can adversely affect the audience’s judgment, indicating that the topic must be written in positive sentences. After selecting a topic, the introduction and overall discussion outline can be pre-organized by completing a topic analysis table.

Students who encounter new formats are often confused, but it is easy to understand if you regard writing a point analysis table as laying the foundation for the introduction. This is not much different from seeing it as an essay written in the order of introduction, main sections, and conclusion. According to the textbook used in Sookmyung Women’s University’s “Critical Thinking and Discussion” class [6], the point analysis table comprises four stages: “need for a new policy—limitations of current policy—possibility of problem-solving—cost comparison.”

### 3.3. Education on Introductory Writing

The first point of introductory writing is the order. Just as there is an order in the four fundamental arithmetic operations, the order of the content delivery can decide whether it is logical or illogical, and how well one sequences one’s arguments is a determining factor between winning or losing in a debate contest. This is similar to the concepts of dividing “assertions” and “basis” from elementary, middle, and high school. Looking back on the arguments written, students often confuse “assertions” and “basis.”

From the students’ data, it is clear that they are already fully aware of the topic and receive counseling at a clinic with some background knowledge on the discussion. Therefore, the instructor encourages students to make good use of what they have already studied. When guiding the students, if the instructor tells them what to say from their studies and research first and last, this could determine the fate of the contest and allow the students to shake off their fear before the debate. How can we more effectively present our findings in writing? Considering that the introduction is based on three grounds for an argument, the first sentence of each argument becomes a topic sentence. The sentence after the topic sentence is a basis that adheres to the truth. If these rules are well-respected and reflected, the sentences form a more systematic flow.

The second point is to divide the categories by the argument. These can be divided by the scale (such as the personal, corporate/institutional, or social/national) or by the field (such as the economic, political, or environmental). It cannot be stressed enough how important it is to consider and divide the arguments by the hierarchy of uniformity or differentiation. If you divide arguments and evidence (which are the first point) and then divide them by categories, you can see the results of uncertain evidence finding its proper place. Additionally, the teachers train the students to write their pros in a direction that emphasizes the cultivation of anticipated effects when adopting the thesis. At the same time, while those on the opposition team are encouraged to write an argument focusing on the harmful effects that may occur if the thesis is adopted. This is summarized in Table 3.

### 3.4. Confirmation of Questions at the Clinic and Cross-Examination Training

#### 3.4.1. Educational Case

In the clinic on the confirmation of questions, the students’ confirmation questions were evaluated and analyzed using Thyer’s “Steps of Critical Thinking Process” [17]. Table 4 provides detailed education examples related to confirmation questions conducted at the Sookmyung Women’s University’s Institute ODC.

#### 3.4.2. Three Steps of the Confirmation Questions

Confirmation questions through critical thinking are in the same context as splitting questions, and taking the time for confirmation questions is the same as managing time to debunk the other person’s logic. The principle of the confirmation question is in the form of a closed question, which is a question method that induces a yes or no answer. Students are instructed to ask one confirmation question at a time over several stages. If the questions are split, the loopholes in their logic become evident.

Step 1 is a step of verifying the confirmation questions from the content of the other party’s introduction. This step aims to ensure that the opponents have accurately heard the arguments and induce a reasonable answer by hinting at the content of the question. It must be done with the facts mentioned by the other party. The second stage is a stage of targeting the opponent’s loopholes and verifying their logic by using contrasting examples of facts (contrasting to the other party’s introduction) mentioned in the first stage. Among the three stages, the logic of the questioner is the most explicitly revealed in the third step of the confirmation questions. A contrasting case can be used, and the validity of their argument can be questioned. The third stage is to expand loopholes, find hidden premises, target them, and reverse the facts promoted by the opponents in the first stage. Hidden premises or errors can be found by specifying the opponent’s logical loopholes and asking questions. There is also no need to lead confirmation questions to assert an opinion. This is only a step for verification, and the debate mentors teach their students to make self-assertions in the introduction and forget about it for the rest of the discussion.

In this example, the topic of scientific discussion is “Animal testing should be prohibited.” and “The Paris Climate Change Agreement is effective.” Table 5 below provides detailed education examples related to confirmation questions before and after the clinic.

### 3.5. Discussion Attitudes and Quasi-Verbal and Non-Verbal Communications

College students who are not familiar with speaking in public find it difficult to ask or present questions. Particularly in South Korean society, where discussion culture is not established, students may find it more difficult to logically and formally hold a discussion. Therefore, students need to practice so that they become used to the tone of the discussion and acquire a basic discussion formality.

Words should be selected according to the discourse of the discussion while respecting the formality of the discussion. The attitude that fits the formality of the discussion emphasized in this discussion clinic is as follows:

1. When it comes to discussions, a particular style of tone is used in South Korea (e.g., instead of saying “hae-yo,” which is a casual tone, South Koreas use “seup-ni-da,” which is more formal).
2. In the beginning, all speakers must reveal which side they are supporting and which part they are starting with (e.g., this is the start of the introduction for agreement).
3. When all speakers finish their speech, as in the beginning, they must clarify which side they are on and which part is the end (e.g., this is the end of the argument for agreement).

Additionally, verbal, non-verbal, and quasi-verbal communication are critical in discussions. When students are guided to use all the above communication methods, this can create a synergy effect. Students should also carefully utilize non-verbal content [19], such as the appropriate use of terms and honorifics, the volume and the speed of the voice, clear and concise pronunciation, considering the audience, delivering accurate linguistic content, speaking passionately, good posture and wearing presentable clothes, using appropriate body language, and effectively using the given space. Coaching body language, namely movement, posture, gestures, impressions, and facial expressions, is equally important. The tone, speed, height, and pronunciation corresponding to the semi-verbal (anti-verbal) expressions in the discussion are also essential elements of a successful discussion.

### 3.6. Online Clinic

The ODC was conducted online, and the current zoom online meetings had a very positive effect during COVID-19. As online discussion clinics are not limited by time and space, they can solve practical problems that face-to-face clinics used to have, such as securing space and time. Discussion clinic experts can provide materials to students directly through hyperlinks, and students can cross-check their materials with the same team, which makes it easier to proceed with the discussion. As it is easy to provide and share related materials through online clinics, this is an advantage that offsets the limitations of online discussion forums.

The advantages of online clinics were further exhibited through the scientific issues in individual online discussion clinics. This is because scientific issues require more rapid and detailed explanations of related theories and research progress, visual presentation of data, and sharing of vast amounts of data than other disciplines. Online clinics make this easier by providing hyperlinks and utilizing social network platforms and cloud storage systems. Additionally, scientific issues often require a multidisciplinary approach related to social background, which makes online platforms more effective.

When it comes to scientific issues, it is more important to display data than to explain theories with words. The ODC was very helpful in providing experimental images, data, and video, such as environmental maps. This demonstrates that a science-themed discussion clinic is effective in exploring science topics. It is a practical education technique, and the spirit of scientific inquiry can be further expressed because students are given specific instructions on how to explore scientific concepts and find relevant information.

### 3.7. Discussion on Scientific Issues

Compared to issues from other disciplines, scientific discussions require social background and anthropological thinking. Subsequently, convergent thinking can be developed through discussion education, and the thinking process of a discussion clinic such as the one explored in this study can help promote scientific thinking. Scientific inquiry methods and critical thinking processes of discussion are similar. The critical thinking process, in which the same remarks are considered for an in-depth analysis, is quite similar to the scientific inquiry method that increases the objectivity and validity of scientific inquiry after multiple trials. The attitude of logically exploring the evidence is similar to debate clinics, which further encourages students to explore scientific validity and objectivity. This is because discussion classes are 90% preparation and 10% performance, which helps students naturally acquire the spirit of scientific inquiry during the preparation time. Critical thinking processes through discussion training can improve objectivity and validity. The discussion process to verify validity is difficult to acquire only through classes, and as 90% of the preparation process can be made up of discussions, clinic education can systematically improve the scientific inquiry spirit. Therefore, discussion clinics can help cultivate a spirit of scientific inquiry.

The importance of discussion can improve how future generations approach science and technology. Writing and speaking are related to human literacy, and critical thinking skills obtained through discussion require a basis in the study of humanity. Reflecting on science and technology anthropologically through critical thinking is vital to prevent science and technology from becoming catastrophic for humanity. Furthermore, it is necessary to consider the need for convergent thinking in the scientific realm ahead of the Fourth Revolution. New concerns that have never existed before will be brought up in social and ethical aspects such as laws and institutions.

It is now an era in which not only a handful of experts need scientific knowledge and technology to express, convey, and communicate scientific knowledge, but the public now also acquires this knowledge. Information on science and technology is separated from the public, and scientific research papers are difficult to comprehend as they are full of jargon, and sometimes it is difficult for even scientists to understand. The former Editor-in-chief of the US journal *Science*, Donald Kennedy, pointed out problems that are difficult to understand even among scientists, with increasingly specialized research methodologies and data analysis graphs as scientific research digs into detailed topics. If the language of science is so esoteric and specialized, the decisions of related policies can also be misleading [19] (p. 715). Neurobiologists from the Karolinska Institute in Sweden have compared and analyzed 70,000 abstracts of biomedical papers since 1881 and published articles proving that scientific papers are becoming less readable on the biological database website BioArchive [20]. This article demonstrates that more journalists, policymakers, and broader public readers should write to be understood. The same applies to scientists as they can encounter obstacles in verifying the reproducibility of research results because of difficult language.

## 4. Conclusions

Among the contents of the discussion clinic, the two discussion topics related to scientific issues included “Animal testing should be prohibited” and “Paris Climate Change Agreement is effective.” Based on these two discussion topics, this paper demonstrated educational case studies on discussion teaching content, an educational model, and the argument analysis used in the discussion clinic counsel built on the basis of Toulmin’s Good Reason Theory on Critical Thinking Education [15] and Thyer’s Development of the Critical Thinking Teaching Resource [17]. This article designed an educational model using their theories as a methodology and aims to devise an innovative form that can teach critical thinking and decisions online.

To understand the needs of the students, their application responses were systematically analyzed. In the survey results, students wanted help with the preparation process necessary for the discussion and the practical strategies for facilitating discussions. They wanted personalized counseling demonstrating that discussion education provided in the foundational curriculum is insufficient. Sookmyung Women’s University ODC could effectively extend these areas through individual discussion counseling rather than lecture-style discussion education.

Proficiency in debates is not a natural talent but is deeply related to acquiring procedural knowledge that gradually develops through ongoing critical thinking training. In other words, if one repeatedly practices for a long time, one’s discussion skills can eventually improve. Through the discussion clinic, discussion skills can be prepared and trained much more effectively by understanding the process and principles of discussion centering on the components of public speaking. In the current lecture-style university education courses, there are many difficulties not found in the parallel of discussion education and implementation. This is because what one says in real-life discussions is essential, and theoretical education alone is difficult to assist students in acquiring practical skills. Discussion clinics are an effective way to compensate for the shortcomings of this lecture-style discussion education. Even if the content is excellent, it can be ineffective if the content cannot be understood and applied in one’s speech. Students need to consider how they want to express their logical thinking process using their language and how they want to arrange their studies logically.

From the analysis of the ODC, it was evident that the structure of the speech was a problem, and by providing technical tips on the speech structure, the students’ fear and frustration about the discussion were resolved. Even with a good textbook, reading what is written in the textbook is insufficient, and individual differences are not considered in the regular curriculum, making it impossible to provide customized education to each student. Therefore, one or two direct consultations (clinics) are much more effective.

Many people assume that the exchange of mutual feelings is limited in online discussion platforms compared to face-to-face classes, but in regular open lectures that follow the standard teaching curriculum, the exchange of personal feelings, values, and experiences is more limited. This indicates that online clinics might be a better approach to performing effective comparative programs as we can always manage the time online to provide personal feedback. Students and instructors can communicate and exchange emotions more effectively through individual customized counseling.

According to Lee and Shin’s [21] survey results, students have positively evaluated replaying recorded videos several times to help them understand the content. They demonstrated that online discussion feedback systems could be an effective teaching tool for students to understand educational content and correct their problems because they can interact in Zoom and watch content repeatedly through recorded videos.

Academic discussions at universities revolve around practical teaching methods and conducting academic discussions among the students. The competencies that can be obtained from academic discussions are linked to the increased readability of academic writing. The ability to effectively deliver complex messages to others is a key competency demanded in the 21st century.

Through individual discussion counseling such as discussion clinics, students can develop their ability to critically examine knowledge and information, learn how to convey their intentions, and acquire practical communication skills.

Communication skills are important for all university majors, but practice is needed to improve the ability to effectively deliver easy-to-understand messages, especially for students majoring in science. 

Kachenstein et al. [22] researched e-learning classes in “Zukunftslabor Gesundheit” (ZLG) due to the need for a new educational concept to convey knowledge to society to provide goal-oriented online education. In their article, they argued that experts lost trust in the context of COVID-19 pandemic management and provided room for misinformation.

Although the ability to write and speak is becoming more essential through discussion education in scientific issues, academic readability is failing because oversimplified explanations outside science attract audiences, and distorted and simplified versions of science replace the truth and threaten academia. The importance of communication skills in the field of science is significantly heightened because it is necessary to observe, think, understand, and infer scientific principles and phenomena and effectively convey them.

The spreading of distorted facts in science is a concern that demonstrates that it is essential to improve communication skills to convey accurate scientific facts and persuade the readers legitimately. Scientists are often not trained to speak or write, and discussion clinics offer comparative programs that are as relevant as the common essential subjects in liberal arts and general education and are linked to major classes, which can help science major students improve writing and speaking skills. As there are relatively few opportunities to develop these abilities as part of a science major compared to other departments, it is insufficient to only take these courses as a compulsory foundational elective.

Despite the growing importance of communication skills in science, discussion clinics can be an alternative when there is no way to cultivate these communication skills through the subjects. Including discussion classes as part of a foundational general education course to teach first-year university students to make presentations for university classes and become familiar with class discussions is restrictive as it cannot cater to students’ needs with different majors. Discussion clinics can provide customized education for first-year students taking discussion courses and all grades connected to their majors. They can also be effectively used to improve scientific communication skills, especially for future scientists.

As revealed in the climate agreements and animal testing issues covered in this analysis, scientific issues include discussions on problems created by science and technology and technology utilization plans and ethical standards. In the process of accurately, objectively, and logically delivering information and knowledge, communicating through public speaking and securing social justification for the development of technology need to be effective. When scientific discourse is entrenched in the way it talks about or understands a particular reality, and when any discovery or innovation occurs, society relies on the initial explanation of concepts provided in the scientist’s language [23]. As Reeves [24] (p. 184) said, “When science speaks, we pay attention.” Subsequently, how a scientist delivers a message is critical for society. Online discussion clinics are suitable for dealing with scientific issues that need quick responses to changes and immediate reactions to new technologies. They provide timely feedback in more diversified ways.

Additionally, many changes follow discoveries and technological innovations that require social consensus. The importance of discussion education will increase in the future as it determines the direction of scientific research at present. How these discussion clinics are conducted through online feedback systems can provide an alternative to communication education methods corresponding to the changing times.

In this article, an educational analysis of an ODC consisting of discussion topics on scientific issues was conducted. Individual counseling was carried out based on the discussion of scientific issues at the ODC, and the findings confirmed that the ODC helped students improve their thinking and communication skills on scientific issues. The advantages of online clinics were further exhibited through the scientific issues in individual online discussion clinics. This is because scientific issues require more rapid and detailed explanations of related theories and research progress, visual presentation of data, and sharing of vast amounts of data than other disciplines. Online clinics make this easier by providing hyperlinks and utilizing social network platforms and cloud storage systems.

The ODC, which was conducted online, provided effective education to students. As there are no time and space restrictions, the online discussion clinic has the advantage of solving practical problems such as securing space and time that face-to-face clinics had, easily providing and sharing data.

In science, which requires faster and more detailed explanations of related theories and research progress than in other fields, visual presentation of data, and massive data sharing, online clinics were able to provide technical support.

This study examined the effectiveness of the ODC’s program for students and presented an educational model that can teach critical thinking online. Based on the analysis of educational content that provided discussion counseling on scientific issues to students, it was confirmed that discussion education is necessary to resolve the disconnection of communication, which is considered a problem of today’s scientific research.

Although the number of participants in the survey or training is small, the experience of operating the ODC confirms the need for online discussion counseling. It expects more participants and data to increase if the ODC system is more stable and lasts longer. The researchers of this paper will continue to track this through follow-up studies. Online education complements face-to-face teaching, enabling a new concept of learning for a generation of digital native students.

## Figures and Tables

**Table 1:** The clinic area and contents of the counseling and guidance.

Clinic Area	Contents of Counseling and Guidance	Clinic Area	Contents of Counseling and Guidance
Preparation of Discussion	Research materials Analyze discussion points Complete the discussion briefs Complete the introduction	Facilitation of Discussion	Make strategies for both for/against Learn methods for confirming questions Strategies for counterargument

**Table 2:** Online Discussion Clinic sections preferred by the students.

		Reaction		Case Percentage
		N	Percentage	
Preferred Debate Clinic	Introduction	34	28.6%	59.6%
	Point analysis	22	18.5%	38.6%
	Discussion outline	21	17.6%	36.8%
	Argument cards	10	8.4%	17.5%
	Data research and organization	7	5.9%	12.3%
	Discussion facilitation	24	20.2%	42.1%
	Others	1	0.8%	1.8%
Total		119	100.0%	208.8%

**Table 3:** Introduction clinic before and after.

	Students’ Introductory Statement before the Clinic	Students’ Introductory Statement after the Clinic	Focus of the Clinic
Animal testing should be prohibited (Agree)	It can prevent infringement of animal rights.	- It can protect the rights of animals. - Prohibiting animal testing can prevent numerous side effects caused by animal testing. - Even if animal testing is prohibited, there are plenty of ways to replace animal testing.	(1) Rather than first revealing and emphasizing the problem, change your perspective toward claiming the expected effect. If the expected effect is first mentioned in the first sentence (assertion), and a problem is presented as a basis, it can be a more convincing argument. (2) Differentiate arguments in terms of various categories. Balanced arguments can be presented, and through this, questions and answers can be approached in various ways to improve logic.
	Prohibiting animal testing can prevent numerous side effects caused by animal testing.		
	Even if animal testing is prohibited, alternatives to animal testing can be used thanks to the development of current technology.		
Animal testing should be prohibited (Disagree)	Animal testing can save other lives, including human lives.	- If animal testing is prohibited, it could disrupt the driving force behind the development of new drugs and vaccines. - If animal testing is prohibited, it becomes unclear how to prove the stability of new drugs.	(1) Prepare the argument centered on harm. If you write an argument in the first sentence of the body paragraph and present a problem as a basis, it can be a more convincing argument. (2) Differentiate arguments in terms of various categories. Balanced arguments can be presented, and through this, questions and answers can be approached in various ways to improve logic.
	Humans and animals are biologically similar.		
	Animal testing is the most effective method.		
The Paris Climate Change Convention is effective. (Agree)	The Paris Climate Change Convention promised investment in renewable technology to improve environmental problems.	- The Paris Climate Change Convention is effective in reducing greenhouse gas emissions. - The Paris Climate Change Convention embodies its goals through direct and specific interventions. - The Paris Climate Change Convention is an international mechanism for responding to environmental disasters.	(1) Rather than first revealing and emphasizing the problem, change your perspective toward claiming the expected effect. If the expected effect is mentioned first in the topic sentence (assertion), and a problem is presented as a basis, it can be a more convincing argument. (2) Differentiate arguments in terms of various categories. Present balanced arguments, and through this, questions and answers can be approached in various ways to improve logic.
	The Paris Climate Change Convention works.		
	The Paris Climate Change Convention aims at a discriminatory principle of responsibility.		
The Paris Climate Change Convention is effective. (Disagree)	The Paris Climate Change Convention is an ineffective agreement.	- The effect of suppressing greenhouse gas emissions is insignificant, and there is a risk of economic loss. - The power play within the agreement can be induced, and political discrepancy and tension can occur in that the internal situation varies from country to country. - It confirms contradictions in environmental ethics.	(1) Prepare the argument by centering on harm. If you write the topic sentence of the argument paragraph and present a problem as a basis, it can be a more convincing argument. (2) Differentiate arguments in terms of various categories. Present balanced arguments, and through this, questions and answers can be approached in various ways to improve logic.
	The Paris Climate Change Convention might be politically harmful.		
	You can see the contradictions in the ethical aspects across different countries from the Paris Climate Change Convention.		

**Table 4:** Educational cases of “Confirmation Question” using the steps of critical thinking process [17].

Critical Thinking Stages	Explanation	Confirmation Questions	A Confirmation Question That the “Agree” Team Can Ask the “Disagree” Team	A Confirmation Question That the “Disagree” Team Can Ask the “Agree” Team
Observation	- Determine what information you can acquire. - Collect information from various sources. - Check the information that already exists. - Explore different perspectives/views - Identify commonalities and contradictions.	- How was it possible to acquire information on the other party’s arguments or objections? - Is it possible from a different perspective? - Is there anything to refute or contradict?	- Using the difference in opinions between China and the US, the US announced that their carbon emissions decreased even after their withdrawal from the Paris Convention, and China claims that their emission level increased. Is that correct?	- Since the adoption of the Paris Climate Change Convention, the prediction that this agreement will work because countries across the globe will reduce greenhouse gas emissions remains conditional depending on the actions taken by each country. Do you think goals can lead to desired results in real life?
Analysis	- Organize information into the main topic or argument.	- Is this what the opponent means when you summarize their arguments or objections?	- Currently, due to the nature of the country, China’s industry is operating without considering various environmental aspects. Do you agree on this part?	- Is it correct to compare the Kyoto Protocol with the Paris Climate Change Agreement and assert that the Paris Convention has expanded its boundary to include Kyoto as well?
Evaluation	- Distinguish the value of the information. - Identify priorities with important information. - Distinguish between opinions and facts.	- If so, is the basis for the introductory argument so important? - Which is more important?	- In other words, China is facing a problem arising from the characteristics of its own national policy, and technically, is this unrelated to joining the Paris Convention?	- Is the Paris Climate Agreement accountable for the demolition of the world’s largest coal company? Is it not because of the development of alternative energy?
Questioning	- Consider other possible alternatives. - Create a new hypothesis.	- Ask for another alternative. - Ask for a new hypothesis.	- Do you admit that it is hypocritical to simply discuss the effectiveness of the Paris Convention while failing to address the difference between China and the US?	- As an example of support for developing countries for raising national competency, it is said that the World Bank invests a lot of money in projects to reduce carbon emissions in developing countries, which is not just an investment, but a bond that holds the recipient countries accountable. Is that right?
Contextualization	- Contextualizing information based on the following criteria: history, ethics, politics, culture, environment, and other specific situations.	- How do you mean this from a historical perspective? - How do you mean this from an ethical perspective? - How do you mean this from a cultural perspective? Continue to apply different areas of discipline for contextualization.	- The US unilaterally withdrew from the Paris Agreement and said they expected the promise to support developing countries would also be compromised. Is that right?	- Do you agree that this creates a relationship between creditors and debtors and is no different from taking on debt based on the standards of global institutions that exercise their dominance over developing countries?
Self-reflection	- Create questions and experiments about conclusions. - Reflect on possible results.	- Does the result of the introduction lead to this reflection?	- Do you admit that it is an error of hasty generalization to be convinced that other developed countries will also cut off support just because the US broke its promise to support developing countries?	- Do you agree that World Bank investment is not a pure investment and can place great pressure on developing countries to join international politics in a pretense exchange for national autonomy?
Discussion Topic: “Animal testing should be prohibited”				
Critical Thinking Stages	Explanation	Confirmation Questions	A confirmation question that the “agree” team can ask the “disagree” team	A confirmation question that the “disagree” team can ask the “agree” team
Observation	- Determine what information you can acquire. - Collect information from various sources. - Check the validity of the collected information. - Explore different perspectives/views. - Identify commonalities and contradictions.	- How was it possible to acquire information on the opponent’s arguments or objections? - Is it possible from a different perspective? - Is there anything to refute or contradict?	- Do you agree that animal testing is a life-killing experiment? - Is it true that animal testing is of practical help to human testing?	- All animal experiments will be conducted only after the Animal Ethics Council has thoroughly reviewed the 3R rules. Do you agree?
Analysis	- Organize information into the main theme or argument.	- Is this what the opposition team means when you summarize their arguments or objections?	- The animal experiment is said to be the way to save the lives of humans and other creatures. Is that right?	- Despite inflicting pain on living creatures, the animal experiment was still approved because the Animal Ethics Council also recognized the legitimacy and necessity of this experiment. Do you agree?
Evaluation	- Distinguish the value of each information. - Identify priorities with important information. - Distinguish between opinions and facts.	- If so, is the basis for the introductory argument so important? - Which is more important?	- Do you admit that animal testing is an experiment that takes away more lives before saving other lives?	- Do you admit that animal testing that is verified through the Animal Ethics Council does not violate ethical aspects?
Questioning	- Consider other possible alternatives. - Create a new hypothesis.	- Ask for another alternative. - Ask for a new hypothesis.	- Do you admit that the percentage of diseases shared by humans and animals is only 1.16%, and there is only a 10% chance that humans will have the same results?	- Do you admit that the data accumulated so far through animal experiments in the past made it possible to develop current technologies for the alternatives?
Contextualization	- Contextualizing information based on the following criteria: history, ethics, politics, culture, environment, and other specific situations.	- How do you mean this from a historical perspective? - How do you mean this from an ethical perspective? - How do you mean this from a cultural perspective? Continue to apply different areas of discipline for contextualization.	- Do you admit that animal testing is inefficient compared to the number of life losses from an economical perspective?	- Do you agree that it is difficult to ban animal testing to develop alternative technology?
Self-reflection	- Create questions and experiments about conclusions. - Reflect on possible results.	- Does the result of the introduction lead to this reflection?	- Do you agree that the opposition’s statement that animal testing is helpful for human experiments is a rushed generalization and hence a systematic error?	- Do you agree that animal experiments can provide safety and be beneficial? - Do you admit that there is a loophole in the propositional argument that animal testing is meaningless?

**Table 5:** The three steps of the confirmation questions before and after the clinic.

	Students’ Confirmation Questions before the Clinic	Focus of the Clinic Confirmation Questions	Students’ Confirmation Questions after the Clinic
Animal testing should be prohibited. (Agree)	You said that animal experiments could save the lives of humans and other creatures, but is it right to save lives at the expense of countless creatures’ sacrifices and deaths?	Instruct one confirmation question to be asked in at least three different stages like below. Step 1: Check if you have listened to the other person properly and imply your understanding of the content of the question. - Method: “On the opposing side, you said A is B. Is that right?” Step 2: Point out problems and target the opponent’s logical loopholes. Present an example that contrasts with the example used as a fact by the opponents in step 1 and ask if their argument is still valid. - Method: “However, according to C, A may not be B or A may be C. Do you agree/acknowledge this?” Step 3: Find a hidden premise, attack it, and reverse the facts in Step 1. - Method: “Do you agree/acknowledge that A is not necessarily B?”	1-1. You said that animal experiments could save the lives of humanity and other creatures. Is that right? 1-2. Do you agree that animal testing is an experiment that involves killing a life? 1-3. Do you agree that animal testing is an experiment that takes away more lives before saving lives?
	2. Is it a selfish human way of thinking to continue conducting animal experiments that already have inaccuracies simply because they may be helpful to humans?		2-1. You said that animal testing is of practical help (provide numerical data sample) for human testing. Is that right? 2-2. However, the statistics indicate that the percentage of diseases shared by humans and animals is 1.16%, and among those diseases, only 10% of humans are likely to have the same results as animals. Do you admit that animal testing can be inefficient compared to the number of lives lost? 2-3. Therefore, do you admit that the opposition’s argument that animal testing is of practical help to human experiments is a hasty generalization error?
	3. Our generation is developing many alternative technologies to replace animal testing. Is it outdated to ignore the effectiveness and possibility of these substitutes and continue animal testing?		3-1. You said that animal testing should continue for humans (or that it is irreplaceable). Is that right? 3-2. However, do you agree that many alternative technologies (simplified in the case of introduction) can replace some animal testing? 3-3. Do you admit that continuing animal testing ignoring the effectiveness and possibility of alternatives can be considered outdated?
Animal testing should be prohibited. (Disagree)	1-1. You said animals are living beings who can equally feel pain, but humans are also living beings with pain receptors. If there is one medicine that can immediately reduce pain, would you like to administer it to animals or humans? 1-2. If you must choose only one living being, do you agree that you would put humans before animals?	Instruct one confirmation question to be asked in at least three different stages like below. Step 1: Check if you have listened to the other person properly and imply your understanding of the content of the question. - Method: “On the opposing side, you said A is B. Is that right?” Step 2: Point out problems and target the opponent’s logical loopholes. Present an example that contrasts with the example used as a fact by the opponents in step 1 and ask if their argument is still valid. - Method: “However, according to C, A may not be B or A may be C. Do you agree/acknowledge this?” Step 3: Find a hidden premise, attack it, and reverse the facts in Step 1. - Method: “Do you agree/acknowledge that A is not necessarily B?”	1-1 You said animal testing was unethical because more than 70% of animal experiments involve pain. Is that right? 1-2. All animal experiments will be conducted only after the Animal Ethics Council has thoroughly reviewed them according to the 3R rules. In other words, animal experiments involving pain were approved because the Animal Ethics Council also recognized the legitimacy and necessity of the experiment. Do you agree? 1-3. Do you admit that animal testing through the Animal Ethics Council does not violate the agreed scope of ethical aspects?
	2. You said that it is meaningless to conduct animal experiments to test a chemical substance that is fatal to humans but not harmful to animals. Then, do you disregard the significance of widely used medicine that went through animal testing to prove that it is safe to be used? Does it mean that animal testing is not meaningless when you reverse this logic?		2-1. You said that it is meaningless to conduct animal experiments if we are testing a chemical substance that is fatal to humans but not harmful to animals. Is that true? 2-2. If the medicine is proven safe to use after animal testing, do you agree that the medicine can be used widely for greater causes? 2-3. Do you admit that there is a loophole in the propositional argument that animal testing is meaningless?
	3. Alternative experimental methods also have errors, and artificial skin or cell multiplication has its own limitations because they are not real human skin and not real human cells. Can you argue that such an alternative technology is good enough to replace animal testing?		3-1. You said animal testing should be prohibited and replaced by the technological development of alternative tests. Is that correct? 3-2. Do you admit that the data accumulated so far through animal experiments in the past made it possible to develop current alternative technologies? 3-3. Do you then agree that it is difficult to ban animal testing even for the advancement of alternative technologies?
The Paris Climate Change Agreement is effective. (Agree)	1-1 You mentioned the difference between China and the US and that the level of carbon emissions in the States has decreased even though the US has withdrawn from the Paris Convention. Is that right? 1-2. You said that China’s carbon emissions increased despite being included in the Paris Climate Agreement, but is this also correct?	Instruct one confirmation question to be asked in at least three different stages like below. Step 1: Check if you have listened to the other person properly and imply your understanding of the content of the question. - Method: “On the opposing side, you said A is B. Is that right?” Step 2: Point out problems and target the opponent’s logical loopholes. Present an example that contrasts with the example used as a fact by the opponents in step 1 and ask if their argument is still valid. - Method: “However, according to C, A may not be B or A may be C. Do you agree/acknowledge this?” Step 3: Find a hidden premise, attack it, and reverse the facts in Step 1. - Method: “Do you agree/acknowledge that A is not necessarily B?”	1-1. You cited the difference between China and the US, saying that despite the US withdrawal from the Paris Convention and China’s participation in the Agreement, the levels of carbon emissions decreased in the US, whereas they increased in China. Is that right? 1-2. Currently, China’s industry operates without considering environmental sustainability due to national policies. Do you agree with this? 1-3. China is facing a problem arising from national policy, and this is a separate matter to joining the Paris Convention. Subsequently, it is contradictory to discuss the effectiveness of the Paris Convention by simply referring to the statistical difference between China and the US. Do you agree?
	2-1. The US unilaterally withdrew from the agreement and said it expected the convention’s promise of supporting developing countries to be broken. Is that correct? 2-2. Then, do you think developed countries are doing others a favor by promising to support developing countries? Or is it their duty to help them? 2-3. You said the US is taking an eco-friendly step to reduce carbon emissions after withdrawing from the Convention. Then, do you think they are fulfilling their responsibilities?		2-1. The US unilaterally withdrew from the agreement and said it expected the promise of supporting developing countries to be destroyed. Is that true? 2-2. The US is not the only country that promised to support developing countries. Do you agree? 2-3. You are certain that the US’s decision to breach the agreement in support of developing countries would affect other developed countries to cut their support. Do you acknowledge that this is a mistaken judgment call made from a hasty generalization?
The Paris Climate Change Agreement is effective. (Disagree)	1-1 Comparing the Kyoto Protocol with the Paris Convention, you mentioned that the Paris Convention expanded the applicable countries to all relevant parties from the Kyoto Protocol. Is that correct? 1-2. You said this fact guarantees the effectiveness of the Paris Convention. Is that right? 1-3. Do you mean that quantitative increases guarantee qualitative increases? 1-4. Before we move on to the next question, I want to point out that this is committing an inconsistent error in failing to identify a specific causal relationship between the premise and the conclusion.	Instruct one confirmation question to be asked in at least three different stages, as below.Step 1: Check if you have listened to the other person properly and imply your understanding of the content of the question. - Method: “On the opposing side, you said A is B. Is that right?” Step 2: Point out problems and target the opponent’s logical loopholes. Present an example that contrasts with the example used as a fact by the opponents in step 1 and ask if their argument is still valid. - Method: “However, according to C, A may not be B or A may be C. Do you agree/acknowledge this?” Step 3: Find a hidden premise, attack it, and reverse the facts in Step 1. - Method: “Do you agree/acknowledge that A is not necessarily B?”	1-1. Comparing the Kyoto Protocol with the Paris Convention, you claimed that the Paris Convention expanded the applicable countries to all relevant parties from the Kyoto Protocol. Is that correct? 1-2. Do you agree that quantitative increases do not guarantee qualitative increases? 1-3. Do you admit that your stated facts are inconsistent with providing a specific causal relationship between the premise and the conclusion?
	2-1. Regarding the measures to reduce greenhouse gases, you mentioned that developed countries need to support developing countries in the following areas: financial help, technology transfer, and nurturing capacities. Is that true? 2-2. If so, developing countries are objects that simply accept the financial resources, technology, and capacity training from developed countries. Is that right? 2-3 If so, the Paris Convention creates a skewed power play within the international community because of the contents of the Convention. Is that right? 2-4. Then, the power dynamics, which had not been highlighted before, will rise to the surface. Do you agree? 2-5. Our agreement team would like to point out the bigger problem that the implementation of the Paris Climate Agreement will cause. It will give powerful authority and advantages to certain individual countries in the international community and act as a negotiating card.		2-1 You mentioned that financial resources, technology transfer, and capacity training from developed countries to developing countries could be used to reduce greenhouse gases. Is that right? 2-2. Then, do you admit that developing countries would become objects that simply accept resources, technology transfer, and capacity training from developed countries, which can lead to a power play within the international community? 2-3. Do you agree that the Paris Climate Agreement’s implementation processes for reducing greenhouse gasses will provide a powerful advantage to certain individual countries in the international community and act as a negotiating card?
	3-1 You took the World Bank as an example of support for developing countries to cultivate competency. Is that right? 3-2. You said that the World Bank invests more money into projects to reduce carbon emissions. Is that right? 3-3. You mentioned that this investment is not just an investment but a bond that expects a return at the end. Is that correct? 3-4. If the opposition calls for the implementation of the Paris Convention’s true efforts, the World Bank’s investment should remain as an investment, and the relationship between investors and local business operators should be different from the relationship between creditors and debtors. Is that true? 3-5. We can deduce that the Paris Convention will eventually be a political or economic negotiation card by indebting the developing countries using the standards of a global organization (the World Bank). This method of pretending to give developing countries sovereignty but forcing them to enter international politics where they would always be indebted is something to be reflected upon.		3-1. As an example of support for developing countries to cultivate their capabilities, you said that the World Bank invests a lot of money in projects to reduce carbon emissions in these countries. However, this is a bond that is expected to be returned and not a simple investment; is that true? 3-2. Do you agree that it is no different from inducing a relationship between creditors and debtors as developed countries use the standards of globally influential organizations (such as the World Bank) to indebt developing countries? 3-3. In other words, do you agree that the World Bank’s investment is not a pure investment and can be a way to pressure the developing countries to join international politics while pretending to give them autonomy?
